# INVITED PAPERS

**DOI:** 10.1038/sj.bjc.6601054

**Published:** 2003-06-27


**British Journal of Cancer (2003) 88**(Suppl 1), S9 – S10
© 2003 Cancer Research UK All rights reserved 0007 – 0920/03 $25.00
**www.bjcancer.com**

**INVITED PAPERS**

**CT 1**

*NEAT* (NATIONAL EPIRUBICIN ADJUVANT TRIAL) AND SCTBG BR9601 (SCOTTISH CANCER TRIALS BREAST GROUP) PHASE III ADJUVANT BREAST TRIALS SHOW A SURVIVAL ADVANTAGE FOR SEQUENTIAL ECMF.

Christopher J. Poole*, Helena M. Earl, Janet A. Dunn, Louise Hiller, Sarah Bathers, David Spooner, Robert J. Grieve, Rajiv K. Agrawal, Liz Foster, Chris Twelves on behalf of the *NEAT* Investigators and the SCTBG. Cancer Research UK Clinical Trials Unit, Institute for Cancer Studies, Edgbaston, Birmingham

***NEAT*** and **SCTBG BR9601** address the role of Epirubicin in adjuvant chemotherapy for women with early breast cancer. ***NEAT*** (2021 eligible patients) compared Epirubicin (100mg/m$^2$ x4cycles) followed by classical (c) CMF (x4 cycles) with cCMF (x6 cycles); **SCTBG BR9601** (370 eligible patients) compared Epirubicin (100mg/m$^2$ x4 cycles) followed by iv dose modified 3-weekly CMF (750:50:600 x4 cycles) with iv 3-weekly CMF (x8cycles). Prognostic characteristics were balanced across treatments: 72% were node positive; 59% ≤50 years old; 58% of tumours grade 3; 57% >2cms; 32% ER-ve, 50% ER+ve (18%NK). For the more intensive cCMF-containing ***NEAT*** regimens, median delivered dose intensity was 93%/course, with similar myelosuppression, amenorrhoea, supportive treatments, treatment-related mortality rates and global Quality of Life.

The first pre-planned joint interim analysis is based on 309 deaths/428 relapses or deaths (median follow-up 32 months (IQR21–45)). Despite lower than anticipated rates in the control arm, there is a highly significant benefit in favour of ECMF for both RFS (HR 0.70, 95%CI 0.58–0.85; p=0.0003) and OS (HR 0.64, 95%CI 0.51–0.81; p=0.0001) irrespective of trial, lymph node status, age and ER status. Both treatment regimens are tolerable, with achievable optimal dose intensities. These data add to those of the Overview in respect of an anthracycline advantage and augur for ECMF as an established standard adjuvant therapy.

**CT2**

RANDOMISED TRIAL OF PACLITAXEL IN COMBINATION WITH PLATINUM CHEMOTHERAPY VERSUS PLATINUM-BASED CHEMOTHERAPY IN THE TREATMENT OF RELAPSED OVARIAN CANCER (ICON4/OVAR 2.2) Jonathan A Ledermann*, on behalf of ICON and AGO Collaborators, MRC Clinical Trials Unit, 222 Euston Road, London NW1 2DA, UK

Patients (pts) with ovarian cancer relapsing ≥ 6 months after platinum therapy are generally retreated with platinum-based chemotherapy. ICON4 (co-ordinated by MRC and Mario Negri Institute (IRFMN)) and OVAR 2.2 (co-ordinated by the German AGO group) were parallel trials comparing 6 cycles of platinum chemotherapy (Plat) versus paclitaxel plus Plat (Pac-Plat) in pts relapsing with a treatment-free interval of ≥ 6 months (MRC/AGO), or ≥ 12 months (IRFMN). Protocol doses were: paclitaxel, 175mg/m$^2$ or 185mg/m$^2$; carboplatin, minimum AUC 5;cisplatin alone, 75 mg/m$^2$; cisplatin in combination, 50mg/m$^2$. 802 patients from UK, Italy, Norway, Germany and Switzerland were randomised between 01/96 and 03/02. Data were analysed as a single trial stratified by randomising group (MRC, IRFMN,AGO). Pt characteristics were similar both across the randomising groups and treatment arms. By10/02, with a median follow-up of 34 months, 674 (84%) pts had progressed or died, with a hazard ratio (HR) of 0.77 in favour of Pac-Plat (p=0.006). This translates into an absolute improvement in 1-year progression-free survival (PFS) of 9% (40% to 49%; 95% confidence interval (CI) 4%-15%). 463 (58%) pts have died; HR ratio is 0.77 in favour of Pac-Plat (p=0.006), which translates into an absolute improvement in2-year survival of 9% (50% to 59%; 95% CI 3%-14%). There wasno evidence that the effect of Pac-Plat is larger or smaller in any subgroups. These results suggest that Pac-Plat improves survival and PFS in pts with 'platinum-sensitive' relapsed ovarian cancer.

**CT3**

PERIOPERATIVE CHEMOTHERAPY IN OPERABLE GASTRIC AND LOWER OESOPHAGEAL CANCER: A RANDOMISED, CONTROLLED TRIAL (THE MAGIC TRIAL, ISRCTN 93793971). W Allum*, D Cunningham, S Weeden on behalf of the UK NCRI Upper GI Clinical Studies Group. *Epsom General Hospital.

MAGIC was designed to determine whether Epirubicin, Cisplatin and infused 5-FU (ECF) improves survival in operable oesophagogastric cancer.

Patients with resectable oesophagogastric adenocarcinoma were randomised to receive perioperative chemotherapy (CSC arm) or surgery alone (S arm). In the CSC arm, chemotherapy consisted of three pre- and three post-operative cycles, 3 weeks apart, of E 50 mg/m$^2$ IVbolus, C 60mg/m$^2$ in fusion and 5-FU 200mg/m$^2$/day continuous infusion.

Between 1994 and 2002, 503 patients (250 CSC, 253 S) were randomised; 74% were gastric, 15% oesophago-gastric junction and 11% oesophageal cancers. In the CSC arm, 88% of patients completed pre-operative chemotherapy, 55% commenced post-operative chemotherapy and 40% completed all 6 cycles.

Resection was considered curative in 79% CSC compared with 69% S patients (p=0.02, $\chi^2$ test). Post-operative complications were similar (CSC 46%, S 46%) as were deaths within 30 days (CSC 6%, S 7%). Maximum diameter of the resected tumour was 3cm for CSC patients and 5cm for S patients (p<0.001, Mann-Whitney U test). For gastric and junctional patients, pathological staging demonstrated 54% CSC were T1/T2 compared with 36% S (p=0.01, $\chi^2$ test) and 80% CSC were N0/N1 compared with 71% S (p=0.10, $\chi^2$ test).

Thus, ECF produces an increased curative resection rate, reflecting a downsizing and histopathologic downstaging effect. By April 2003, the number of events required for the primary outcome analysis of overallsurvival will be available.

**CT4**

CHEMOTHERAPY AT STANDARD OR INCREASED DOSE INTENSITY IN PATIENTS WITH OPERABLE OSTEOSARCOMA OF THE EXTREMITY: A RANDOMISED CONTROLLED TRIAL (ISRCTN 86294690). Ian Lewis*, Marianne Nooij on behalf of the European Osteosarcoma Intergroup (EOI). *St James's University Hospital, Leeds.

Previous EOI randomised trials have shown that a two-drug chemotherapy regimen of cisplatin and doxorubicin is an effective and tolerable treatment for patients with operable osteosarcoma. The aim of this trial was to investigate whether increasing the dose intensity of this regimen using granulocyte colony-stimulating factor (G-CSF) would improve survival in this rare disease.

Previously untreated patients aged 40 or under with biopsy-proven, non-metastatic osteosarcoma of an extremity were eligible for this trial. Patients were randomised between the standard two-drug regimen and the same regimen intensified by the addition of G-CSF. In the standard arm, chemotherapy consisted of six three-weekly cycles of cisplatin (100mg/m$^2$ 24-hour infusion) and doxorubicin (25mg/m$^2$/day by 4-hour infusion for three days). The doses of cisplatin and doxorubicin were identical in the intensified arm, but cycles were given every two weeks intensified by G-CSF(5mcg/kg/day subcutaneous injection on days 4-13 of the cycle). Surgery was scheduled for week 6 in both arms.

Between May 1993 and September 2002, 504 patients (251 standard arm, 253 intensified) were randomised from centres in Europe, South America, South Africa, Canada and Saudi Arabia. Thus this trial is one of the largest conducted in osteosarcoma. Analysisis planned for Spring 2003 at which time the number of events required for the primary outcome analysis of overall survival will be available.

### CT5

MRC AML 12 - PAEDIATRICS.

Brenda E Gibson*, David Webb, Keith Wheatley, an behalf of MRC CLWP. Siebold de Graaf on behalf of DCOG
*Royal Hospital for Sick Children, Yorkhill, Glasgow G3 8SJ

MRC AML 12 recruited 525 children between 1995 and 2002. It adopted the template of its preceding trial, MRC AML 10, which provided information allowing risk stratification based on cytogenetics and response to initial therapy. Good risk patients – t(8;21), inv(16) or t(15;17) – were not eligible for allograft and no patients received ABMT. Children were first randomised to receive induction chemotherapy with either ADE or MAE and thereafter randomised to receive 4 or 5 courses of treatment in total, where the extra course was HD Ara-C and Asparaginase. If a donor was available, a sibling allograft (n=43) was recommended as the final course.

The CR rate is 92%, induction deaths 4% and 4% showing resistance disease. Deaths in first remission are 6% and the 5 year relapse rate is 33%. There are no significant differences between children receiving ADE or MAE and no advantage for five blocks of treatment over four. Within risk groups survival from CR is superior to that of AML 10 which cannot be explained by the randomised comparisons. 43 children have received an alloBMT, and donor versus no donor comparisons within the standard and poor risk groups do not show any survival differences, although numbers are small.

Risk group stratification based on cytogenetics continues to predict outcome. The limit to which conventional chemotherapy, both during induction and consolidation, can further improve outcome may have been reached and new approaches need to be explored.

### CT6

RANDOMISED TRIAL OF IFOSFAMIDE, CARBOPLATIN AND ETOPOSIDE WITH MID-CYCLE VINCRISTINE (ICE-V) VERSUS STANDARD CHEMOTHERAPY (C) IN PATIENTS WITH SMALL CELL LUNG CANCER (SCLC) AND GOOD PERFORMANCE STATUS (PS) (MRC LU21) P. Clark, on behalf of LU21 collaborators, MRC Clinical Trials Unit, UK

Patients with SCLC and good PS should be offered intensive treatment aimed at prolonging survival and achieving cures. Etoposide, vincristine, ifosfamide, and carboplatin have been shown to be active. MRC LU21 trial is comparing 6 cycles of ICE-V (day 1: i.v. ifosfamide 5 g/m$^2$, carboplatin 300 mg/m$^2$, etoposide 120 mg/m$^2$; day 2: i.v. etoposide 120 mg/m$^2$; day 3: oral etoposide 240 mg/m$^2$; day 14: i.v. vincristine 1.0 mg/m$^2$), at 4-week intervals, versus 6 cycles of C at 3-week intervals. Recommended C regimens were: CDE (day 1: i.v. cyclophosphamide 1 g/m$^2$, doxorubicin 40 mg/m$^2$, etoposide 120 mg/m$^2$; day 2: i.v. etoposide 120 mg/m$^2$; day 3: oral etoposide 240 mg/m$^2$), and PE (day 1: i.v. cisplatin 80 mg/m$^2$, etoposide 120 mg/m$^2$; day 2: i.v. etoposide 120 mg/m$^2$; day 3: oral etoposide 240 mg/m$^2$). 402 (202 ICE-V, 200 C) patients were randomized. Patients characteristics were well balanced; median age 62 years, WHO PS 0-1 in 87%, limited disease in 87% of patients. 74% ICE-V and 83% C patients completed 4 or more cycles of chemotherapy. ICE-V compared with C patients experienced similar moderate or severe alopecia, lethargy, breathlessness, anorexia, and nausea; less mucositis, but more septicemia and bleeding. Response rates for ICE-V and C were 83% and 81%. 307 (76%) patients have died. The HR for survival was 0.77 (95% CI 0.62, 0.97, p = 0.026,) in favor of ICE-V. Median survival was 15.1 (ICE-V) and 11.6 (C) months, and 1-year survival rates were 54% and 45%. In summary, ICE-V improved survival compared with standard chemotherapy in patients with SCLC and good PS with acceptable toxicity.

© 2003 Cancer Research UK

